# No evidence for an association between the -36A>C phospholamban gene polymorphism and a worse prognosis in heart failure

**DOI:** 10.1186/1471-2261-9-33

**Published:** 2009-07-28

**Authors:** Diogo GB Santos, Alessandra Medeiros, Patrícia C Brum, José G Mill, Alfredo J Mansur, José E Krieger, Alexandre C Pereira

**Affiliations:** 1 Laboratory of Genetics and Molecular Cardiology, Heart Institute (InCor), Sao Paulo University Medical School, Sao Paulo, Brazil; 2 School of Physical Education and Sport, University of São Paulo, Sao Paulo, Brazil; 3 Department of Physiology, Federal University of Espirito Santo, Vitoria, Brazil

## Abstract

**Background:**

In Brazil, heart failure leads to approximately 25,000 deaths per year. Abnormal calcium handling is a hallmark of heart failure and changes in genes encoding for proteins involved in the re-uptake of calcium might harbor mutations leading to inherited cardiomyopathies. Phospholamban (PLN) plays a prime role in cardiac contractility and relaxation and mutations in the gene encoding PLN have been associated with dilated cardiomyopathy. In this study, our objective was to determine the presence of the -36A>C alteration in PLN gene in a Brazilian population of individuals with HF and to test whether this alteration is associated with heart failure or with a worse prognosis of patients with HF.

**Methods:**

We genotyped a cohort of 881 patients with HF and 1259 individuals from a cohort of individuals from the general population for the alteration -36A>C in the PLN gene. Allele and genotype frequencies were compared between groups (patients and control). In addition, frequencies or mean values of different phenotypes associated with cardiovascular disease were compared between genotypic groups. Finally, patients were prospectively followed-up for death incidence and genotypes for the -36A>C were compared regarding mortality incidence in HF patients.

**Results:**

No significant association was found between the study polymorphism and HF in our population. In addition, no association between PLN -36A>C polymorphism and demographic, clinical and functional characteristics and mortality incidence in this sample of HF patients was observed.

**Conclusion:**

Our data do not support a role for the PLN -36A>C alteration in modulating the heart failure phenotype, including its clinical course, in humans.

## Background

Congestive heart failure resulting from cardiomyopathies is a serious malady and a leading cause of human morbidity and mortality[1]. In Brazil, heart failure (HF) is responsible for approximately 25,000 deaths per year [2] and is associated with considerable morbidity, since patients with HF undergo frequent hospital readmissions, which are higher in those of lower socioeconomic strata.

Although HF is a common endpoint for many forms of cardiovascular diseases, one of the most common cause of congestive heart failure is the dilated cardiomyopathy (DC), affecting 40 people in every 100,000 of the population [3]. DC is characterized by ventricular chamber enlargement and systolic dysfunction with normal left ventricular (LV) wall thickness [4]. DC may be inherited as a dominant disorder associated with a range of already described genetic defects [5]. Despite genetic heterogeneity, abnormal calcium handling is a hallmark of DC [6]. Therefore, changes in genes encoding for proteins involved in the re-uptake of calcium might harbor mutations leading to cardiomyopathies [7,8].

Phospholamban (PLN) plays a prime role in cardiac contractility and relaxation [9] and mutations in the gene encoding for PLN have been associated with DC. Moreover, specific genotype-phenotype associations may predicted by PLN mutations [10-13].

The genetic variant (A>C) at position AF177763. **1**:g.203A>C (at -36 bp relative to the PLN transcriptional start site: -36A>C) of the PLN gene has contradictory findings in the literature. The first study to describe it [8], found an allelic frequency of 0.6% in DC patients and 2.5% in healthy control individuals. Furthermore, after a functional *in vitro *assay development, the authors did not find an altered transcription activity rate associated with the presence of any particular allele. In contrast, Haghighi et al [14], found this alteration in an allelic frequency of 2.9% and 0.2% in DC patients and in healthy control individuals, respectively. In this later study, they suggested, after *in vitro *functional assays, that this transition might contribute to depressed contractility and accelerated functional deterioration in heart failure.

In this study, our objective was to determine the presence of the -36A>C alteration in the PLN gene in a Brazilian population of individuals with HF and to test whether this alteration is associated with a worse prognosis leading to increased structural heart modifications and a worse prognosis in patients with HF.

## Methods

### Study design

Patients from an ongoing inception cohort of patients with heart failure were included in the present study between 1995 and 2005, submitted to sequential echocardiographic evaluation and to clinical follow-up for death and/or heart transplantation. Eight hundred and eighty-five patients were studied in the General Outpatient Clinic of the Heart Institute of São Paulo University Medical School. The diagnosis of heart failure was made according to previously published criteria [15]. The classification of the etiology of heart failure followed previous recommendations [16,17].

### Inclusion criteria

Patients with symptomatic heart failure of different etiologies and left ventricular ejection fraction ≤ 45% on two-dimensional transthoracic Doppler echocardiography were eligible for the study. Patients were also evaluated for surgical treatment of heart failure, including heart transplantation. Specifically, patients with heart failure due to valve disease enrolled in the study were those with severe left ventricular dysfunction to the point that they would not be eligible for valve repair or replacement, but rather candidates for heart transplantation.

### Exclusion criteria

Patients with heart failure due to valve disease that would be candidates for conventional surgical treatment, such as valve repair or replacement; patients with hypertrophic cardiomyopathy, chronic obstructive pulmonary disease, recent myocardial infarction and unstable angina were excluded. In addition, patients with severe renal or hepatic dysfunction, severe peripheral artery disease, cerebrovascular disease, active infection, coexisting neoplasm and active peptic ulcer disease were also excluded [18].

### Control population – General population of Vitória/ES, Brazil

A cross-sectional study of risk factors for cardiovascular diseases was performed in the urban population of Vitoria, Brazil, using the WHO-MONICA project guidelines. The study design was based on cross-sectional research methodology and was developed by means of surveying and analyzing socioeconomic and health data in a probabilistic sample of residents aged 25 to 64 years from the municipality of Vitoria, ES, Brazil. The population was randomized and the sample was socioeconomically, geographically and demographically representative of the residents of this municipality. A selection of 2,268 residential homes located in Vitoria was made and these were visited. The project received approval from the Ethics Committee of the Biomedical Center of Universidade Federal do Espírito Santo (UFES). The selected individuals were asked to attend the Cardiovascular Investigation Clinic of the University Hospital for tests to be performed on the following day. Of the total sample, 1,577 individuals attended. Participants were submitted to physical examination. Major cardiovascular risk factors such as smoking habits, alcohol intake, sedentarism, diabetes and hypertension were inquired. Blood glucose, total cholesterol, lipoprotein fractions, and triglycerides were assayed by standard techniques in a 12 hour fasting blood sample[19].

### Left ventricular function assessment

Sequential left ventricular ejection fraction was determined by M-mode echocardiography. In some patients two-dimensional method was employed, using Simpson. The evaluation of the left ventricular function was performed by the echocardiography staff in a blinded way in relation to the genotypes and was conducted according to previously published recommendations [20].

### Genotype determination

Extraction of genomic DNA was performed from leukocytes separated from whole blood using a standard method [21]. The genomic reference with GenBank accession number AF177763.1 [GeneBank: AF177763.1] was used to retrieve the PLN sequence corresponding to proximal promoter and exon 1. The -36A>C alteration was detected by a polymerase chain reaction (PCR) technique. We used a web program  to design the forward primer 5'-ATGTGACATCATAAGACCTCCCT**C**G-3' with 1 mismatch nucleotide (in bold – at position -38 of the gene) that abolish a *Taq*I restriction site in the mutated sequence. The reverse primer 5'-ATTTCAAAGTTGTCTCATT-3' was designed using the PrimerSelect program (Lasergene^®^). The digested fragments by *Taq*I were undertaken in a blinded way after the samples had been separated with electrophoresis on a 3% agarose gel and stained with ethidium bromide (1 μg/ml).

### Statistical analysis

Data are presented as means ± SDs for continuous variables and as frequencies for categorical variables. Differences in baseline characteristics among groups were analyzed using Student's *t *test for continuous variables and the Chi-square test for categorical variables. Survival curves were calculated for each genotype with the Kaplan-Meier method, and differences between the curves were evaluated with the log-rank statistic. Multivariable tests were performed if an association was detected adjusting for possible confounders. The risk for events was expressed as an odds ratio with 95% confidence intervals. Statistical analyses were performed with SPSS software. A p value < 0.05 was considered significant.

### Ethics

The study protocol was approved by the Ethics Committee for Medical Research on Human Beings of the *Hospital das Clínicas *from University of São Paulo Medical School. Signed informed consent was obtained from all participants.

## Results

After genotyping 881 patients with HF and 1259 general control individuals we found the alteration in 51 HF patients (three homozygotes) and 63 individuals from the general population (two homozygotes), because of the small number of homozygote individuals, they were joined with heterozygote for further analysis. Allelic frequency was 3,1% in HF patients and 2,5% in the general control sample. Genotypic frequencies were not significantly different when comparing HF and normal individuals (p = 0,50).

Analyzing only individuals with HF, the comparison of baseline demographic and clinical characteristics did not reveal any statistically significant difference between the genotype groups (Table 1). The same is seen for the general control population, with the exception of ethnicity (Table 2).

**Table 1:** Clinical and demographic characteristics of the HF sample according to genotype.

	AA	AC + CC	P value
Number	830	51	-

Age, years	52.74 ± 14.76	56.16 ± 13.33	-

Gender			
Male(%)	67.63	64.71	0.66

Ethnicity (%)			0.86
Blacks	95.5	4.5	
Mulattos	95.5	4.5	
Whites	93.6	6.4	
Others	100	0	

Etiology (%)			0.55
Chagasic	92.9	7.1	
Idiopathic	95.7	4.3	
Hypertensive	95.9	4.1	
Ischemic	93.6	6.4	
HF due to valve disease	91.1	8.9	
Others	93.9	6.1	

Biochemical			
Serum sodium (mg/dL)	136.79 ± 4.49	135.90 ± 4.84	0.28
Hemoglobin (mg/dL)	13.07 ± 2.14	13.03 ± 2.07	0.90
Total Cholesterol (mg/dL)	192.03 ± 50.51	192.76 ± 46.43	0.93
Triglycerides (mg/dL)	126.05 ± 76.44	149.42 ± 86.34	0.72
HDL (mg/dL)	44.09 ± 14.57	44.49 ± 15.93	0.87
LDL (mg/dL)	121.31 ± 42.62	118.89 ± 38.91	0.74
Creatinin (mg/dL)	1.32 ± 0.65	1.48 ± 0.52	0.09
Glycemia (mg/dL)	112.78 ± 54.36	114.79 ± 50.68	0.81

BMI (kg/m^2^)	25.36 ± 4.98	26.2 ± 4.97	0.34

Heart Rate (pm)	79.73 ± 13.16	79.38 ± 11.01	0.61

Diastolic Blood Pressure (mmHg)	76.26 ± 18.57	73.66 ± 13.7	0.45

Systolic Blood Pressure (mmHg)	122.12 ± 31.36	118.97 ± 24.83	0.59

**Table 2:** Clinical and demographic characteristics for the general control population sample according to genotype.

	AA	AC + CC	P value
Number	1198	61	-

Age, years	45 ± 11	44 ± 11	-

Gender			
Male (%)	45.4	45.9	0.94

Ethnicity (%)			0.006
Blacks	100	0	
Mulattos	96.8	3.2	
Whites	92.6	7.4	
Others	93.8	6.3	

Biochemical			
Total Cholesterol (mg/dL)	205.82 ± 89.68	199.7 ± 63.74	0.47
Triglycerides (mg/dL)	118.5 ± 96.15	110.62 ± 79.23	0.46
HDL (mg/dL)	59.84 ± 36.92	60.66 ± 38.68	0.87
Creatinin (mg/dL)	1.89 ± 2.11	1.93 ± 2,12	0.88
Glycemia (mg/dL)	93.32 ± 39.12	86.72 ± 30.72	0.11

BMI (kg/m^2^)	26.37 ± 4.93	27.16 ± 6.38	0.34

Heart Rate (pm)	70.96 ± 11.26	72.59 ± 9	0.48

Diastolic Blood Pressure (mmHg)	83.39 ± 13.43	82.13 ± 12.43	0.44

Systolic Blood Pressure (mmHg)	127.07 ± 21.99	124.05 ± 18.37	0.22

Next, we tested the association between -36A>C genotypes and functional characteristics acquired through baseline echocardiography examination and, again, we did not observe any significant association for the HF and the general control population. The data are shown in tables 3 and 4, respectively.

**Table 3:** Functional data for the HF sample according to genotype

	AA	AC + CC	P Value
Interventricular septum, mm	9.03 ± 1.98	9.42 ± 2.19	0.24

LV posterior wall, mm	8.93 ± 1.73	9.42 ± 2.22	0.14

LV diastolic diameter, mm	67.52 ± 12.06	65.4 ± 10.47	0.18

LV systolic diameter, mm	57.13 ± 13.64	55.1 ± 12.62	0.33

LV ejection fraction, (%)	40.43 ± 14.87	44.7 ± 15.78	0.08

Aortic diameter, mm	32.91 ± 5.03	34.5 ± 6.03	0.88

LA diameter, mm	46.95 ± 8.56	45.40 ± 7.73	0.19

RV diameter, mm	26.52 ± 7.05	25.03 ± 8.57	0.35

LV mass, g	251.2 ± 93.5	264.4 ± 106.3	0.53

LV – left ventricle, LA – left atrium, RV – right ventricle

**Table 4:** Functional data for the general control population sample according to genotype

	AA	AC + CC	P Value
Interventricular septum, mm	8.94 ± 1.12	9.08 ± 1.29	0.58

LV posterior wall, mm	8.8 ± 1.07	8.9 ± 1.1	0.68

LV diastolic diameter, mm	48.4 ± 4.87	48.89 ± 5.1	0.64

LV systolic diameter, mm	29.02 ± 4.08	29.99 ± 4.37	0.28

LV ejection fraction, (%)	71.35 ± 5.22	69.46 ± 5.78	0.11

Aortic diameter, mm	31.83 ± 2.98	31.25 ± 3.29	0.5

LA diameter, mm	31.83 ± 2.98	31.25 ± 3.29	0.03

RV diameter, mm	17.26 ± 4.65	17 ± 3.60	0.79

LV mass, g	150.6 ± 44	155.6 ± 44,36	0.58

LV – left ventricle, LA – left atrium, RV – right ventricle

Genotypic frequency distribution within the HF sample was not significantly different when comparing different etiologies (Table 5).

**Table 5:** Genotype and allelic frequencies among HF etiologies.

	Genotype Frequency				Allelic Frequency	
	
	AA	AC	CC	P value	A	C
Chagasic	92,9	7,1	0	0.35	96.5	3.5
Idiopathic	95.7	3.2	1.1		97.3	2.7
Hypertensive	95.9	3.6	0.5		95.9	4,1
Ischemic	93.6	6.4	0		96.8	3.2
Valvular	91.1	8.9	0		94.6	4,4
Others	93.3	6.1	0		97	3

No difference was observed in the use of medications for heart failure between genotypic groups at baseline (data not shown), eliminating this as a potential confounder in our longitudinal analysis.

**Figure 1:**
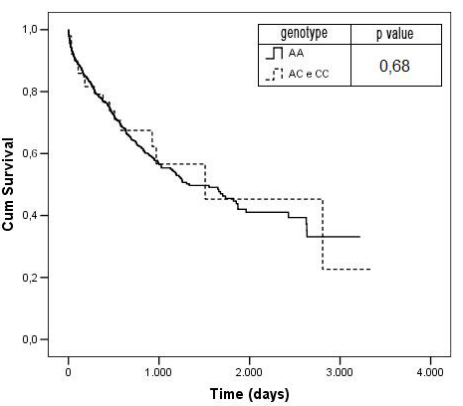
**Kaplan-Meier Mortality curves according to genotype**.

Finally, the mortality or heart transplantation incidence was not different between individuals harboring the allele potentially associated with decreased functional status (Figure 1).

## Discussion

The PLN -36A>C is not a polymorphism described in any database. It was first described in the Spanish population with an allelic frequency of 0,5% (n = 85) in patients with DC and 2,5% (n = 120) in a healthy control population [8]; later it was described in the Greek and Caucasian populations with an allelic frequency of 3,7% (n = 218) and 1,8% (n = 163) in DC patients, respectively. In the Greek population, the variation displayed an allelic frequency of 0,16% (n = 296) in healthy controls [14]. Herein, we have genotyped 881 HF patients, and 1259 general control individuals, the allelic frequencies found were 3,06% and 2,5%, respectively. Comparing the genotype frequencies, we observed that there was no difference between cases and controls studied. In addition, analyzing the allelic frequencies of the different etiologies enrolled, we do not observe any difference between, suggesting there is no specific modulation of this particular genetic variant in any particular etiological process leading to cardiomyopathy.

There are conflicting findings in the literature regarding a potential functional effect of the studied polymorphism. Haghighi et al, have argued that due to the alteration proximity to a putative TATA box (12 nucleotides) it could play a role in gene expression regulation. In their study, they found an increased transcriptional activity and suggested that this was due to the lost of a putative glucocorticoid receptor binding site. On the other hand, Medin et al, 2007, did not find an altered transcriptional activity associated with any particular allele.

Here we have observed a similar genotypic distribution between men and women, and among the different heart failure etiologies suggesting a homogeneous distribution throughout the HF population stratum (Table 1). Likewise, we observed the same homogeneous distribution in our general population sample, data is shown in table 2, excepting for the distribution among ethnic groups where we do not see the presence of the variation in black individuals.

We also observed a lack of association between the polymorphism and clinical (Table 1) and echocardiography (Table 3) data. Again, the same result was found in the general population in table 2 (clinical) and 4 (echocardiography), excepting for the left atrium diameter. However, as this was the unique characteristic with a significant association among nine correlated characteristics with non-significative associations, we believe this is a false-positive finding.

Finally, the Kaplan-Meier survival curves were not significantly different (Figure 1) between groups. This indicated that, despite a potentially functional variant regarding gene transcription rate, no association exists with an increased mortality incidence. All the results previously described were not influenced by a differential use of heart failure medications.

## Conclusion

There is no difference between the frequency of the studied variation between HF and the general population individuals. Taken together, our data do not support a role for the PLN -36A>C alteration in modulating the phenotype or clinical course of heart failure.

## Competing interests

The authors declare that they have no competing interests.

## Authors' contributions

DGBS and AM carried out the molecular genetic studies, statistical analysis and drafted the manuscript. ACP participated in the design of the study, statistical analysis and coordinated experiments and manuscript preparation. PB, JEK participated the design of the study. JGM and AJM were responsible for patient selection and characterization. All authors read and approved the final manuscript.

## Pre-publication history

The pre-publication history for this paper can be accessed here:


